# Multiple brain abscesses caused by *Nocardia farcinica* infection after hand injury: A case report and literature review

**DOI:** 10.1097/MD.0000000000039019

**Published:** 2024-07-19

**Authors:** Kun Xue, Anling Zhang, Shuyu Liu, Dawei Chen

**Affiliations:** a Department of Neuro-oncology Surgery, the First Hospital of Jilin University, Changchun City, Jilin, China; b Department of Stomatology, Jilin FAW General Hospital, Changchun City, Jilin, China.

**Keywords:** brain abscess, central nervous system nocardiosis, mNGS, *Nocardia farcinica*, precise diagnosis, trimethoprim-sulfamethoxazole

## Abstract

**Rationale:**

*Nocardia* infection is commonly regarded as an opportunistic pulmonary pathogen affecting debilitated or immunocompromised individuals. Brain abscesses caused by *Nocardia farcinica* are rare and pose a diagnostic challenge. Traditional diagnostic techniques for identifying *Nocardia* species, such as blood culture, microscopy, and pathology, have shown inadequate performance. In the reported case, we applied metagenomic next-generation sequencing (mNGS) to diagnose a case of brain abscess due to *N. farcinica*.

**Patient concerns:**

A 66-year-old female developed a brain abscess after sustaining a hand injury. The patient exhibited a gradual change in personality and experienced tremors in her right upper limb for a duration of 1 month.

**Diagnoses:**

The pathogen responsible for the multiple brain abscesses was identified in the cerebrospinal fluid as *N. farcinica* through mNGS.

**Interventions:**

Antibiotic treatment included trimethoprim-sulfamethoxazole, linezolid, amikacin, meropenem, and moxifloxacin.

**Outcomes:**

The patient’s symptoms and signs improved significantly after administration of antibiotics to which the pathogen is known to be sensitive. After 5 months of follow-up, magnetic resonance imaging of the head showed that the abscess was basically cured. The patient lived a normal life with no adverse drug reactions.

**Lessons:**

*Nocardia* brain infection is characterized by an insidious onset and lacks distinctive clinical and imaging features. mNGS was advantageous for the timely identification and management of *Nocardia*-associated brain abscess in the present case and obviated the need for invasive brain surgery. Expeditious and precise diagnosis coupled with prompt antibiotic therapy can significantly reduce the mortality rate associated with this condition.

## 1. Introduction

Brain abscesses are an infrequent but severe condition that can afflict immunocompromised individuals and are caused by a diverse array of microorganisms. Nocardiosis, a bacterial infection caused by aerobic Actinobacteria known as *Nocardia*, primarily affects immunocompromised hosts.^[1]^ Inhalation is the typical mode of transmission, and lung involvement is present in over 80% of patients, manifesting as nodules/masses or lung consolidation. Dissemination to other organs occurs in approximately one-third of patients, with a preference for the central nervous system (CNS) as well as skin and soft tissues.^[2]^

Given the potential asymptomatic nature of CNS nocardiosis, it is imperative to conduct brain imaging when there is suspicion or diagnosis of nocardiosis in an immunocompromised patient.^[3]^ However, it is important to note that the imaging characteristics of brain abscesses due to *Nocardia* infection lack specificity and may be mistaken for other infectious or noninfectious conditions that affect immunocompromised individuals.^[4]^ The potential for misdiagnosis and delayed treatment is a notable concern with respect to this condition. In this report, we present a case study involving a patient who developed multiple brain abscesses due to the dissemination of *Nocardia* infection to the CNS following trauma to the hand. The diagnosis was confirmed by the results of metagenomic next-generation sequencing (mNGS), and a combination of antibiotics to which *Nocardia* are sensitive and sequential treatment proved to be effective.

## 2. Case presentation

A 66-year-old female was admitted to the hospital due to change in personality, right upper limb tremor for 1 month and drowsiness for 3 days. The patient had experienced a traumatic hand injury 1 month previously, which healed after she disinfected and dressed the wound herself and took oral antibiotics. She then developed progressive personality changes, including reticence, suspiciousness, irritability, and involuntary shaking of the right upper extremity, and became unconscious 3 days prior to presentation to the hospital.

The patient underwent positron emission tomography (PET)/computed tomography (CT) examination and received symptomatic treatment at a local hospital, and the diagnosis was considered brain metastatic cancer or parasitosis. However, no significant improvement in symptoms occurred, and she was transferred to our hospital on December 7, 2022. The patient had intermittent fever during the course of the disease, with temperature fluctuating between 37.3 and 37.8°C. The patient had no cough or sputum and no history of otitis media, sinusitis, immunodeficiency disease, chronic lung disease, or human immunodeficiency virus (HIV) infection. The patient had been previously healthy, with no personal or family history of atopy.

The results of physical examination included: temperature, 37.3°C; blood pressure, 110/78 mm Hg; heart rate, 87 times/min, and respiratory rate, 20 times/min. The patient was drowsy, opened her eyes when called, but did not respond to questions. Both pupils were equally large and round as well as sensitive to light radiation. She had right upper limb tremor, and her physiological reflexes were diminished. Pathological reflexes were not elicited. No nystagmus was observed, and Kernig sign was positive. Her breathing sounds were clear, and no rales were heard.

**Figure 1.**
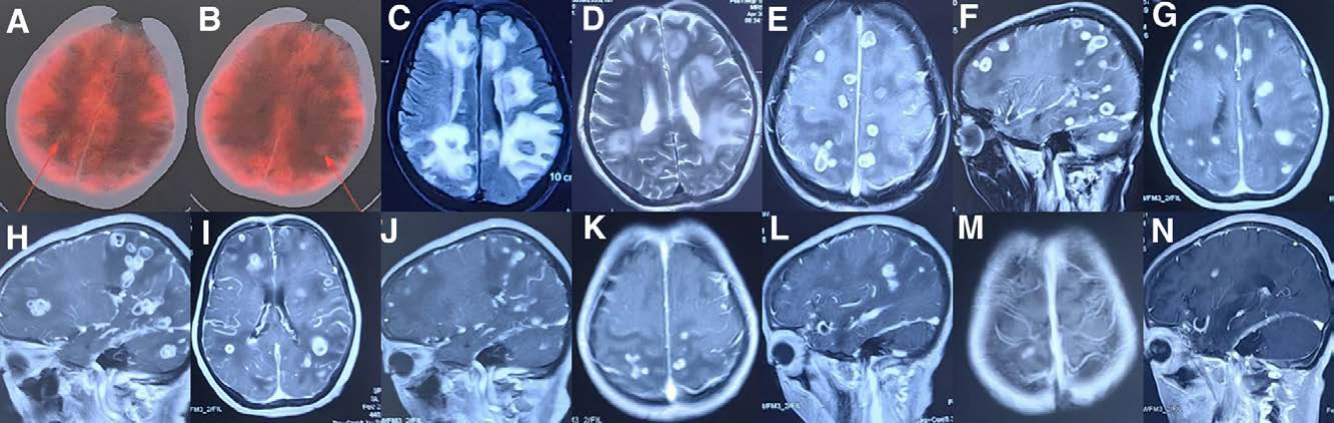
PET/CT showed multiple mixed isointense nodular shadows in the brain parenchyma. The largest one was about 1.5 cm in diameter and partly ring-shaped, with increased FDG metabolism (SUVmax = 5.2, shown by arrow in A–B). MRI of the head showed multiple abnormal signal shadows visible in the cerebral bridge, left hippocampus, right occipital lobe, right corpus callosum, right cerebellum, and bilateral frontal, parietal and temporal lobes, with low signal on T1WI and high signal on T2WI, with obvious irregular ring enhancement and a surrounding edema band (C–F). MRI of the head after 3 wk of treatment showed that the abscess had started to decrease in size (G–H). MRI of the head after 7 wk of treatment showed significant reduction of the abscess (I–J). At the 3-mo follow-up, MRI of the head showed that the abscess was mostly resolved (K–L). After 5 mo of follow-up, MRI of the head showed that the abscess was essentially healed (M–N). CT = computed tomography, FDG = fluorodeoxyglucose, MRI = magnetic resonance imaging, PET = positron emission tomography, T1WI = T1 weighted imaging.

The results of laboratory analysis included: white blood cell (WBC) count, 8.4 × 10^9^ cells/L; neutrophil absolute value, 83.6%; procalcitonin, 0.14 ng/mL; C reactive protein, 3.2 mg/L; erythrocyte sedimentation rate, 19 mm/h; all indicators of liver and kidney function, normal; and blood culture, normal. 1, 3-β-D-glucose test (G test), galactomannan antigen detection (GM test), autoimmunity antibody spectrum, female tumor marker, purified protein derivative test, T-SPOT, and *Brucella* antibody test were all negative. Chest CT findings were normal. PET/CT showed multiple mixed isointense nodular shadows in the brain parenchyma, the largest of which was approximately 1.5 cm in diameter and partly ring-shaped, with increased fluorodeoxyglucose metabolism (SUVmax = 5.2) (Fig. 1A and B). Magnetic resonance imaging (MRI) of the head (2022 Dec 6) showed multiple abnormal signal shadows in the cerebral bridge, left hippocampus, right occipital lobe, right corpus callosum, right cerebellum, and the bilateral frontal, parietal, and temporal lobes. T1 weighted imaging showed a ring-like, slightly low and central low signal, and T2WI showed a ring-central high signal. Fluid attenuated inversion recovery showed isosignal, and after enhancement, there was obvious irregular ring reinforcement with uneven wall reinforcement. The size of the left parietal lesion was 2.0 cm × 1.5 cm, and a band of edema was seen around it (Fig. 1C–F). The admission diagnosis was multiple occupying lesions of the brain parenchyma (nature unknown but possibly representing inflammatory granuloma, brain abscess, metastatic cancer, or parasitic disease).

After admission, the patient was given mannitol (25g every 8 hours, iv) and levetiracetam (500mg, twice a day, oral). Cerebrospinal fluid (CSF) examination revealed a pressure of 180 mmH_2_O, and the CSF was colorless and transparent. Penn test was positive (+). In the CSF, the red blood cell count was 10 × 10^6^ cells/L, and the WBC count was 420 × 10^6^ cells/L. The neutrophil percentage accounted for 84%, with 6% monocytes and normal glucose and chloride levels. The results of bacterial culture, *Tubercle bacillus* detection, Gram staining, acid-fast staining, ink staining, and detection of cryptococcal antigen, mycobacterium, HIV, cytomegalovirus, Epstein-Barr virus, coxsackie virus, human herpes simplex virus-1 (HSV-1, 2), toxoplasma antibodies, and brucellosis antibodies were all negative. mNGS of the CSF revealed *Nocardia* spp. (*Nocardia farcinica*), sequence number 44, relative abundance 10.07%. Sensitivity testing was conducted using the broth microdilution method (BMD) for 15 different antibiotics.^[5–7]^ The results indicated sensitivity to trimethoprim-sulfamethoxazole (TMP-SMX), linezolid, amikacin, meropenem, and moxifloxacin.

Trimethoprim-sulfamethoxazole (TMP-SMX, 1.44 g, 3 times daily, orally), linezolid (0.6 g, 2 times daily, iv), and amikacin (0.5 g, 2 times daily, iv) were given on the same day. After 3 weeks of treatment, the patient temperature had normalized. She regained consciousness, and the tremor of the right upper limb disappeared. Repeat MRI of the head (2022 Dec 29) showed that the abscess had begun to decrease in size (Fig. 1G–H). At that time, her CSF pressure was 150 mmH_2_O, and WBC count was 110 × 10^6^ cells/L, with a neutrophil percentage of 54%, monocyte percentage of 46%, and normal glucose and chloride levels. To avoid the risk of bone marrow suppression, linezolid was replaced with meropenem (2g, 3 times daily, iv). One month later, head MRI (2023 Jan 29) showed that the abscess was significantly reduced (Fig. 1I–J), At that time, her CSF pressure was 130 mmH_2_O, and WBC count was 17 × 10^6^ cells/L, with a neutrophil percentage of 32%, monocyte percentage of 68%, and normal glucose and chloride levels. The patient was clearly conscious, fluent in speech, and cooperative with physical examination. The adjusted treatment plan was oral TMP-SMX (0.96g, 3 times daily), sequential treatment with moxifloxacin (0.4 g, 1 time daily). After 62 days of hospitalization, the patient was discharged and continued treatment for 6 months (adjust the dose according to her renal function and bone marrow suppression). After 3 months of follow-up, head MRI showed that most of the abscess had subsided (Fig. 1K–L). After 5 months of follow-up, MRI of the head showed that the abscess was basically cured (Fig. 1M–N). The patient lived a normal life with no adverse drug reactions.

## 3. Discussion

*Nocardia*, initially reported by Edmond Nocard in 1888, is a prokaryotic bacterium classified under the phylum Thick-walled Bacteria, class Eubacterium, and order Actinomycetes. It is ubiquitously present in various environmental niches, including decaying organisms, seawater, freshwater, dust, soil, and domestic animals.^[8]^ *Nocardia*, as a pathogen with opportunistic tendencies, exhibits an incidence rate ranging from 0.33 to 0.87 per 100,000 individuals, with a higher prevalence in middle-aged and elderly populations, and a male-to-female ratio of 3:1.^[9,10]^ Among the pathogenic strains affecting humans, *N asteroides* accounts for 90% of cases, followed by *N brasiliensis*(7%) and *N caviae* (3%). Notably, *N farcinica*, the most virulent and pathogenic strain, also poses a significant threat.^[11]^ The various modes of infection encompass the respiratory and gastrointestinal tracts, as well as the skin, with respiratory inhalation and trauma serving as the predominant routes of invasion. Among all cases, the incidence of pulmonary *Nocardia* infection ranges from 62% to 86%, while involvement of the CNS occurs in 2% to 26% of cases. Additionally, the cornea and spine may also be affected.^[12]^ Individuals with a compromised immune system are vulnerable to contracting such infections, which include factors such as glucocorticoid usage, malignancy, organ and hematopoietic stem cell transplantation, HIV infection, chronic obstructive pulmonary disease, systemic lupus erythematosus, chronic kidney disease, and other susceptibility factors such as diabetes, alcohol abuse, chronic sarcoidosis, use of tumor necrosis factor-alpha inhibitors, inflammatory bowel disease, and hospital-acquired infections. It is noteworthy that approximately one-third of infected individuals exhibit immunocompetence.^[8,13]^

*Nocardia* brain abscesses account for approximately 2% of all brain abscesses and are mostly solitary, but 80% of brain abscesses caused by *N farcinica* appear as multiple abscesses and have a high mortality rate.^[14–16]^ Patients usually have no obvious symptoms of infection such as fever, and neurological symptoms tend to appear gradually or can progress rapidly. Clinical manifestations include elevated intracranial pressure, impaired consciousness, seizures, and focal neurological deficits. A study of 89 CNS *Nocardia* infections showed that imaging was dominated by circumferentially enhancing lesions (93%), 50% of which showed multiple foci with peripheral edema as a predominant effect.^[9]^ The patient in the present case was in good health, did not use glucocorticoids, did not have underlying lung disease, immune deficiency, exposure to a *Nocardia*-containing environment, or other susceptibility risk factors. The patient had a history of skin trauma, and thus, this case was considered to be a bacterial infection acquired through broken skin and spread to the brain through the bloodstream. Due to the nonspecific clinical presentation and imaging as well as the lack of physician awareness of the disease, such infections are easy to miss or misdiagnose. This case was initially considered as brain metastatic carcinoma or parasitosis at an outside hospital, and the imaging findings must be differentiated from brain abscesses, brain metastatic carcinoma, brain tumors, brain toxoplasmosis, and primary CNS lymphoma caused by common bacteria.^[17]^

The pathogenic detection methods for *Nocardia* include smear, culture, staining, histopathology of lesions, and molecular biology techniques (e.g., 16SrRNA, matrix-assisted laser desorption/ionization time-of-flight mass spectrometry [MALDI-TOF MS]).^[18,19]^ At the same time, the use of empirical broad-spectrum antibiotics has not improved the efficiency of treatment due to the increasing number of clinically resistant organisms. mNGS is the macrogenomic analysis of specimens (blood, sputum, alveolar lavage, CSF, thoracoabdominal fluid, pus and tissue specimens) to obtain biological information such as species classification, drug resistance, and virulence of pathogens (bacteria, viruses, fungi, parasites, mycoplasma and leptospira, etc), and offers timely, extensive and sensitive results.^[20]^ In 2014, Wilson et al first reported detection of intracranial leptospirosis infection by mNGS.^[21]^ mNGS can rapidly and accurately identify microbial species, clarify the pathogenic diagnosis, and guide the timely clinical initiation of goal-directed microbiological therapy, especially for new, acute, and critical infections as well as difficult and complex infections, particularly those for which conventional clinical testing has repeatedly indicated negative findings or unknown pathogens.^[22–24]^ Since mNGS is susceptible to a variety of factors, including sample type, sample processing method, sequencing platform format and strategy, it cannot completely replace traditional methods, but the combination of mNGS with conventional methods can significantly improve clinical diagnostic rates.

In the present case, the results of routine laboratory pathogen examination were negative. CSF culture for 3 days did not clarify the pathogen, whereas the CSF pathogen was clearly identified as *N farcinica* infection by mNGS with negative resistance gene and virulence factor testing, which provided a basis for early targeted antimicrobial drug therapy.

TMP-SMX is the first-line drug for the treatment of *N farcinica*, and the recommended dose is 5 to 10 mg/kg TMP + 25 to 50 mg/kg SMX daily. Other drugs to which *N farcinica* are sensitive include linezolid, imipenem, meropenem, amikacin, ceftriaxone, cefotaxime, ciprofloxacin, and moxifloxacin.^[25]^ Since different *Nocardia* subspecies have different susceptibilities to antibiotics, some have developed resistance to certain antibiotics. The literature reports 42% to 57% resistance to sulforaphane among *Nocardia*, with a gradual increase (85%) among *N farcinica* in particular.^[26]^ Wei et al showed that the sensitivities of 28 strains of *Nocardia* were 100.0%, 100.0%, 92.9%, 75.0%, 67.9%, 67.9%, and 64.3% for TMP-SMX, linezolid, amikacin, imipenem, tobramycin, ceftriaxone, and cefotaxime, respectively.^[15]^ Schlaberg et al analyzed the susceptibility of 1299 *Nocardia* strains and showed that all genera were susceptible to linezolid, 2% were resistant to sulfonamides, and the overall resistance rate to amikacin and imipenem was low.^[27]^ Rahdar et al reported a case of brain abscess caused by *N asteroides* infection in a patient with comorbid diabetes. The patient exhibited resistance to multiple antibiotics. However, after surgical intervention and treatment with imipenem and linezolid, the patient condition significantly improved. Joshua et al reported a case of multiple brain abscesses caused by *N araoensis* infection. Their patient was successfully treated with a 6-week course of oral TMP-SMX combined with meropenem, followed by oral amoxicillin and clavulanate. Barry et al reported a case of multiple brain abscesses due to *N otitidiscaviarum* infection. Despite undergoing neurosurgical intervention and a comprehensive treatment regimen that included TMP-SMX, amikacin, linezolid, moxifloxacin, and doxycycline, the patient unfortunately succumbed to the condition. Meena and colleagues conducted an analysis of 206 cases of CNS nocardiosis. Their findings revealed that 34% of the patients had normal immune function, with the use of corticosteroids being the most prevalent risk factor. The survival rate for brain abscesses was significantly higher with the combination of neurosurgical intervention and treatment with TMP-SMX along with 2 to 3 antibiotics to which the pathogen was sensitive, including amikacin, meropenem, and linezolid. The treatment duration ranged from 6 to 12 months, with an overall mortality rate of 22.8%.^[11,28–30]^

Therefore, identification of *Nocardia* spp. subspecies and analysis of drug resistance genes are needed to improve cure rates and prognosis by following the principles of early, combined, adequate and full course of treatment. In the present case, after 7 weeks of treatment with TMP-SMXcombined with amikacin, linezolid, and meropenem, imaging showed progressive regression of intracranial lesions, and return of the WBC of the CSF to basically normal. No adverse drug reactions were observed over 62 days, and fungal infections were taken into account during treatment for *Nocardia* infection. The clinical pharmacist was involved throughout the treatment process, and the individualized plan was formulated and adjusted for the patient from the pharmacological perspective, which ensured the safety of the patient medication.

Surgery is an indispensable tool in the diagnosis and treatment of *Nocardia* brain abscess, with 18.3% of cases reported in the literature requiring surgery.^[31]^ The 1-year survival rates for surgical and non-surgical patients were reported to be 80% and 33%, respectively, with surgical patients having better outcomes and stereotactic abscess aspiration being preferred.^[32–35]^ For abscesses < 2 cm with no risk of high cranial pressure and brain herniation and stabilized infection for 4 weeks of drug therapy, if the abscess does not shrink, stereotactic abscess puncture should be performed. For abscesses > 2.5 cm, puncture and pus aspiration combined with antibiotic therapy should be performed. Thick-walled, loculated abscesses that continue to increase in size after 2 weeks of drug therapy or for which therapy is ineffective after 4 weeks, as well as abscesses at risk of breaking into the ventricles and cerebellar abscesses causing obstructive hydrocephalus, removal is necessary and will shorten the duration of antibiotic use and reduce the toxicity and economic cost of multidrug therapy. In the present case, the patient had abscesses < 2 cm, and the imaging, CSF indexes, and clinical symptoms all gradually improved on dynamic observation during drug treatment. Therefore, surgery was not performed. The cure rate of Nocardia brain abscesses can reach more than 90% with early diagnosis and treatment, but if the diagnosis is delayed, the morbidity and mortality rate is as high as 50%^[36]^Therefore, early identification of the causative organism and individualized adjustment of the treatment plan according to the disease and treatment outcome are necessary to improve the cure rate and prognosis.

## 4. Conclusions

In conclusion, the reported case suggests the need to be alert to the possibility of *Nocardia* infection in immunocompetent patients with intracranial single/multiple brain abscesses. mNGS can accurately, rapidly, and promptly identify the pathogenic bacteria, avoiding misdiagnosis, missed diagnosis, disease progression, and prolonged treatment. For treatment, TMP-SMX combined with 2 to 3 effective antibiotics with good blood–brain barrier permeability is preferred. The treatment should be adequate, regular, and complete for 9 to 12 months, with dynamic assessment of efficacy and monitoring of drug toxicity, supplemented by surgery if necessary to reduce recurrence and improve the cure rate.

## Author contributions

**Data curation:** Anling Zhang.

**Resources:** Shuyu Liu.

**Writing – original draft:** Kun Xue.

**Writing – review & editing:** Dawei Chen.
